# Patient perspectives on interventional pain management: thematic analysis of a qualitative interview study

**DOI:** 10.1186/s12913-020-05452-7

**Published:** 2020-07-01

**Authors:** Johan Hambraeus, Kjerstin S. Hambraeus, Klas-Göran Sahlen

**Affiliations:** 1 Department of Epidemiology and Global Health, Umeå University, SE90185 Umeå, Sweden; 2 Smartkliniken Eques Indolor, Stolp-Ekeby 24, SE18695 Vallentuna, Sweden

**Keywords:** Interventional pain management, Qualitative study, Thematic analysis, Empowerment, Patient-focused, Chronic pain

## Abstract

**Background:**

Chronic pain is a widespread problem that is usually approached by focusing on its psychological aspects or on trying to reduce the pain from the pain generator. Patients report that they feel responsible for their pain and that they are disempowered and stigmatized because of it. Here, we explored interventional pain management from the patient’s perspective to understand the process better.

**Methods:**

A purposive sample of 19 subjects was interviewed by an independent interviewer. The interviews were transcribed into text and thematic analysis was performed.

**Results:**

The subjects’ perceptions covered three key themes: themselves as objects; the caregivers, including the process of tests and retests, the encounters and interactions with professionals, and the availability of the caregivers; and finally the outcomes, including the results of the tests and treatments and how these inspired them to think of other people with pain. Linking these themes, the subjects reported something best described as “gained empowerment” during interventional pain management; they were feeling heard and seen, they gained knowledge that helped them understand their problem better, they could ask questions and receive answers, and they felt safe and listened to.

**Conclusions:**

Many of the themes evolved in relation to the subjects’ contact with the healthcare services they received, but when the themes were merged and structured into the model, a cohesive pattern of empowerment appeared. If empowerment is a major factor in the positive effects of interventional pain management, it is important to facilitate and not hinder empowerment.

**Trial registration:**

Clinicaltrials.gov 2013-04-24 (Protocol ID SE-Dnr-2012-446-31 M-3, ClinicalTrials ID NCT01838603).

## Background

Pain has been defined as “an unpleasant sensory and emotional experience associated with actual or potential tissue damage, or described in terms of such damage” [1]. This definition implies that pain is modulated by sensory, emotional, and cognitive functions. When pain persists chronically (more than about 3 months), this modulation becomes even more evident [2]. Nearly 20% of the population in Sweden [3] and a similar proportion of people in developing countries are affected by chronic pain, making it a global problem [4, 5]. Pain is a major factor that prompts people to seek help from different healthcare providers [6], and chronic pain is a significant cause of impaired health-related quality of life (HRQoL) [7]. Several epidemiological studies in both developed and developing countries have shown that the prevalence of chronic pain is around 20% in the population [3, 4]. The WHO have shown in the annual Global Burden of Disease study that low back pain has remained the main cause of disability since 1990 [8]. Chronic pain is also one of the most costly diseases, consuming a large part of the health budget both in Sweden and USA [9, 10].

Pain generators (e.g., pain derived from zygapophyseal joints, intervertebral discs, and nerve root entrapments) are important factors in chronic pain, although their exact roles are still obscure [11–14]. It is still controversial whether pain generators are triggers that only initiate the chronic pain or if they are underlying factors in the maintenance of pain.

The healthcare community has approached this problem in different ways. Interventional pain management (IPM) focuses on the pain generators and tries to eliminate them [15, 16], whereas pain rehabilitation programs (PRPs) focus on the cognitive and behavioral aspects of pain and try to reduce the negative consequences of persistent pain [17]. Both strategies are applied to patients with unexplained chronic pain. Over the last decades, the gold standard treatment of non-cancer-related chronic pain in Sweden has been PRPs; IPM has only been used sparingly [18].

The motivation behind this study was hearing that patients with chronic pain describe bad experiences from encounters with healthcare providers [19]. Many qualitative studies have focused on the patients’ experiences of living with chronic pain and have shown that healthcare providers may blame the patients for being responsible for their pain [20] while disempowering the patients [21]. This results in stigmatization of patients with chronic pain [22]. Patients have described how they strive for self-management [23, 24], how they feel distrusted and disrespected when interacting with healthcare providers [25], and how they believe their needs are ignored [26]. Healthcare providers have confirmed this view of patients with chronic pain [27], which is based on feelings of inadequacy in the treatment of pain among some healthcare providers [28]. The challenge faced by healthcare providers during encounters with patients with chronic pain is described by the incompatible requirements of empowering patients [29] while maintaining a professional perspective to avoid disempowering themselves [30, 31]. Chronic pain is often described as a psychological disease, where the initial pain generator triggers sensitization and the patient continues to experience pain despite the trigger having disappeared, and thus chronic pain is treated with psychological methods (e.g., coping and cognitive behavioral therapy) [32, 33]. In contrast, there are the studies that show a reduction of psychological problems and sensitization after treatment of pain generators [12, 14]. Health-related quality of life has been reported to improve significantly after interventional pain management [34], and the improvement is greater than after PRPs, both in magnitude and number of patients who improve [35, 36]. However, we have not found any qualitative studies exploring the patients’ experiences of interventional pain management, and this prompted us to explore the topic.

## Methods

### Aim

The aim of this study was to explore IPM from the patient’s perspective to understand IPM better.

### Study setting

The study was performed at one of the few IPM clinics in Sweden, located in a rural part of Stockholm. There were 159 new patients referred to the clinic and 1456 encounters where nerve blocks were performed in 2017. According to official statistics for 2017, there were 21,083 encounters with pain physicians in Sweden, and 1712 of those encounters included nerve blocks to diagnose and/or treat pain [37], which means that this clinic provided 7% of the encounters and 85% of the nerve blocks performed in Sweden that year. The medical facility is housed in a private residential building and occupies most of the ground floor. The patients have access to the garden outside the building and to a waiting room with freely available coffee. The staff include one physician, one nurse, and one dog trained in animal-assisted therapy for the relief of stress and anxiety. The healthcare provided through the clinic is reimbursed mainly through procured contracts with national health insurance schemes. General practitioners refer patients from all parts of Sweden when conservative therapy with physiotherapy, medications, and coping strategies has failed to give sufficient relief. All clinical procedures are performed according to the Spine Intervention Society Guidelines [38]. IPM mainly focuses on zygapophyseal joint pain, and searching for pain generators by performing blocks of medial branches of cervical, thoracic, and lumbar spinal nerves.

### Subjects

A purposive sample of 20 subjects who had undergone IPM at the clinic was selected to provide a heterogeneous cohort of subjects. The selection was performed by the nurse in February to March, 2017, from among patients who had been treated or were undergoing treatment at the facility. We looked for disparities in age, living conditions, gender, pain localization, and pain duration and for patients with good ability to communicate their feelings and experiences. One subject initially agreed to participate but withdrew at the time of the interview. Therefore, 19 subjects were enrolled with an age range of 25–66 years (Table 1), with 11 women and 8 men. Ten subjects had suffered from pain for 2–6 years, and nine had suffered from pain for 12–40 years before their first visit. All of the subjects were diagnosed with zygapophyseal joint pain and treated with conventional continuous radiofrequency denervation (thermal ablation at 80 °C for 1 min, with several parallel lesions against each nerve) [34], although this was not a criterion for inclusion in the study. At the time of the interviews (April to July, 2017), 11 subjects reported a clinically significant improvement in HRQoL (defined as an improvement of ≥0.1 on the EQ-5D index, calculated from the five dimensions of the Euroqol EQ-5D-3: mobility, self-care, activity, pain, and psychological distress) compared with their first visit to the IPM clinic [39, 40]; five reported unchanged HRQoL; and two reported impaired HRQoL (≥0.1 reduction). The procedures experienced by subjects at our clinic included medial branch blocks; intra-articular injections in joints, including the sacroiliac joint; lateral branch blocks of the sacroiliac joint; radiofrequency denervation of the medial branches (zygapophyseal joints) and lateral branches of the sacroiliac joints; sympathetic nerve blocks; and interlaminar and transforaminal nerve-root blocks (Table 1).

**Table 1:** Description of the subjects in the study

Age (years)	Pain duration (years)	Pain localization	Cause	Previous treatments received	Procedures received on the ward	EQ-5D index change from first visit	Clinically significant change in EQ-5D index (difference > 0.1)
30–40	12	T, L	N	Phys, Med, Fusion	MBB, RF, ia, LBB	+ 0.707	+
> 60	5	H, C	N	Phys, Med, PRP	MBB, RF, IL	+ 0.094	0
40–50	4	C, L	N	Phys, Med, PRP	MBB, RF	−0.069	0
50–60	40	L, O	N	Phys, Med, PRP	MBB, RF, ia	−0.104	–
20–30	2	L	T	Phys, Med	MBB, ia, RF, TF		
50–60	20	C, T, L	T	Phys, Med, PRP, CII, MBB, RF	MBB, RF, ia, LBB, Symp	+ 0.173	+
50–60	30	L	N	Phys, Med, Fusion, DCS, MBB	MBB, RF	+ 0.568	+
50–60	17	H, C, L	R	Phys, Med, TP	MBB, IL, RF, ia	+ 0.258	+
40–50	17	L	N	Phys, Med	MBB, IL, RF, TF, Symp, ia	0.000	0
50–60	15	H, C	R	Phys, Med, PRP, Fusion, TP	MBB, RF	+ 0.167	+
50–60	3	L	N	Phys, Med	MBB, RF	+ 0.263	+
40–50	6	C, T	N	Phys, Med, Fusion, TP	MBB, RF	+ 0.601	+
40–50	2	L	R	Phys, Med, PRP	MBB, ia, RF, Symp	−0.327	–
50–60	3	T	N	Phys, Med, PRP	MBB, RF	+ 0.033	0
40–50	4	L	T	Phys, Med, PRP	MBB, RF, ia	+ 0.264	+
50–60	18	C, T	R	Phys, Med, MBB, pulsed-RF	MBB, RF	+ 0.263	+
50–60	3	H, C, L	N	Phys, Med, MBB, pulsed-RF	MBB, RF	+ 0.071	0
50–60	4	L	N	Phys, Med, PRP	MBB, RF	+ 0.601	+
50–60	25	C, T, L	T	Phys, Med, Fusion	MBB, RF, IL, ia, TF,	+ 0.105	+

*H* headache, *C* cervical pain, *L* lumbar pain, *T* thoracic pain, *O* other pain localizations, *R* road traffic accident, *T* other trauma, *N* no trauma, *Phys* physiotherapy, *Med* medication (usually paracetamol, NSAIDs, opioids, gabapentin, and pregabalin and/or amitriptyline), *PRP* pain rehabilitation program, *MBB* medial branch blocks to diagnose zygapophyseal joint pain, *LBB* lateral branch blocks to diagnose sacroiliac joint pain, *NB* peripheral nerve block, *RF* conventional radiofrequency denervation, *pulsed-RF* nerve stimulation with pulsed radiofrequency current, *IL* interlaminar nerve root block, *TR* transforaminal nerve root block, *Fusion* surgery with vertebral fusion, *DCS* dorsal column stimulation, *ia* intraarticular injections, *CII* continuous intrathecal opioid infusion, *Symp* nerve block of sympathetic nervous system, *TP* trigger point injections

Most of the subjects reported pain affecting several regions of the body (Table 1). The main location of pain was the lumbar region in 15 subjects and the neck in four subjects. The subjects lived in rural or urban environments, with a geographical distribution ranging from the northern to southern parts of Sweden.

All 19 subjects had undergone conservative treatments by primary care physicians and physiotherapy, eight had been through PRPs focused on acceptance and commitment therapy, and 14 had also been assessed by orthopedic surgeons, of which four underwent surgical interventions. Dorsal column stimulation devices were implanted in three subjects, and one subject had an intrathecal opioid infusion device implanted at arrival at the clinic. Four subjects had experience of diagnostic tests, including pulsed radiofrequency nerve stimulation in two subjects, and one had previously undergone radiofrequency denervation (Table 1). All of the subjects said that they hoped to get help with pain relief and that this was the reason for them coming to the clinic.

### Researchers

The research group consisted of three people. JH is a specialist in anesthesiology, intensive care, family medicine, and pain management. He has treated patients with chronic pain in primary care since 1998 and has provided IPM since 2003. KH has 20 years’ experience as nurse in stroke rehabilitation and she has been working in IPM full-time since 2014. K-GS is an experienced qualitative researcher. He has been working as a nurse for many years, but has no experience of working with IPM or chronic pain.

### Pilot study

After ethical approval and obtaining informed consent, a pilot study was performed with seven subjects selected in a similar manner to the study group. KH performed pilot interviews with the subjects in a spare room at the clinic. The interviews were recorded and transcribed. However, several problems with the procedure became apparent. The subjects were happy to be interviewed, but because the nurse was involved in their usual care, the subjects did not distinguish the research interviews from their ordinary healthcare evaluations. Therefore, they found it difficult to focus on the research questions we were interested in, and there was a risk that the subjects left out relevant information because they assumed that their medical history was well known. It also became clear that the interview guide needed to be shorter and more focused.

Another problem was that because we wished to include subjects from all regions of Sweden to reflect our clinical population, it was necessary to conduct the interviews on the same day the subjects visited the clinic for treatments. We could not motivate them to make an extra trip of 200–400 km in some cases just to participate in interviews. The subjects were tired from the care they received that day, which limited their endurance for participating in the interviews.

Based on our experiences in the pilot study, we changed the design of the interviews, including the interview guides (Additional file 1) as follows.

- The questions were reformulated to focus more on the subject’s experiences during IPM and less on their previous experiences.
- The introductory questions were shortened and the interviews started with questions focusing on the first time they visited the IPM clinic.
- We switched from face-to-face interviews to telephone interviews to ensure that the interviews were not limited to subjects living close to the clinic, and the interviews were not performed on the same day that the subjects received care in the clinic.
- An experienced interviewer without previous connection to the clinic was recruited to perform the interviews (see Acknowledgements). She has no competing interests or potential conflicts of interest, she was not involved in the subjects care at the IPM clinic, and she had never met the subjects before she performed the interviews.

After performing three interviews using the revised approach, the researchers listened to the recordings to ensure that the changes in the design provided the intended results and that the interviewer performed as expected.

### Interviews

The subjects selected for the study were informed of the study by the clinic’s nurse. This included information about how the study was to be performed and the aim of the study. The same information was given to the subjects in written form, and they were asked to read the information and provide written informed consent to participate. The subjects were informed that a research assistant (see Acknowledgements) would call them to schedule a recorded telephone interview. The research assistant, who was not involved in the subjects’ care, contacted the subjects, asked again for consent, and scheduled a telephone appointment. The research assistant also instructed the subject to be seated comfortably in a relaxed location where they would be able to talk undisturbed during the telephone interview.

At the scheduled time, the research assistant called the subjects on a telephone line that was recorded. After reconfirming consent, the interview was performed. The questions were intended to help the subjects describe various aspects of their experience of IPM, including what they felt during the encounter at our clinic and their reflections during and after the encounter and procedures (Additional file 1). The interviewer also asked several follow-up questions to help them describe what they had felt and thought, without leading them, and to help them reflect on their experiences and feelings during the IPM program. After the interview ended, the research assistant disconnected the call and stopped the recording. The research assistant then called the subjects 5 min later to allow the subjects to comment on the interview or to withdraw from the study. The subjects were also asked if they would like to add anything or if they had any further questions, and were offered contact with the IPM clinic in case the interview had raised any feelings that they needed help with. The comments were written down by the research assistant. All interviews took place between May and August 2016. Each interview was 13–45 min long. All interviews were performed in Swedish, transcribed into Swedish text, and analyzed. The themes, codes, and citations were translated into English when writing the manuscript.

### Thematic analysis

Thematic analysis was performed as described by Braun and Clarke [41], and is an analytical approach well-suited to exploring subjects’ views. The research group read and familiarized themselves with the transcribed text, and codes were generated for the latent and manifest content. Themes were identified among the codes, and these were changed until consensus was reached. The transcriptions were coded after each interview on a line-by-line basis. We felt that saturation was reached before all the interviews were analyzed because no new codes were identified when coding the last interview. Nevertheless, the analysis continued until all of the interviews were coded [42]. The last interviews to be coded served more as confirmation rather than adding further data. The thematic analysis was based on our common knowledge and experience, and the themes ranged from self-explanatory (e.g., “hope of recovery” or “ambivalence about the future”) to more concrete (e.g., “accept of pain to become pain-free”) to detect manifest and latent content. The themes were discussed and categorized. Open Code 4.03, which is software developed for qualitative research analysis, was used for the thematic analysis [43].

### Triangulation

Triangulation was performed continuously using several approaches, namely, comparing the interviews, comparing how the different researchers interpreted the text, and briefly describing the results to other subjects encountered in the clinic to see if they recognized themselves in the themes. The different pre-understandings the researchers had gained from encounters with subjects in IPM or other healthcare provided a background to the analysis. The results were discussed in the research group until consensus was reached for all themes. The originally transcribed texts were checked frequently to ensure that the themes were applicable. The themes were also checked to see if there were differences by gender or age, or whether the subjects were in a situation where they reported improvements in HRQoL compared with their first visit.

### Validity check

After the analysis, the participating subjects were contacted by the last author (K-GS) who presented them with the results and asked them to elaborate on the results, add information, or clarify their responses.

### Ethical considerations

Ethical approval was obtained from the regional ethics board in Umeå, Sweden (Dnr 2012–446-31 M). The subjects were assured that confidentiality would be maintained and that it would not be possible to identify them individually. They were given written information about the study and provided written informed consent to participate. Informed consent was also explicitly obtained when scheduling the interview, at the start of the interview, and after the interview. The subjects could leave the study at any time, and one subject withdrew from the study after providing written consent at the time of scheduling the interview. The study was registered on Clinicaltrials.gov (Protocol ID SE-Dnr-2012-446-31 M-3, ClinicalTrials ID NCT01838603). The COREQ Equator checklist was used (Supplementary File 1) [37].

## Results

The themes recognized in the interview material covered various factors and were categorized as follows: individually focused themes (intrinsic factors) that were related to and expressed by the subjects themselves; healthcare-focused themes (the importance of care-givers) that focused on the services provided; and outcome-focused themes that focused on the outcomes and goals expressed by the subjects.

### Individually focused themes: intrinsic factors

The intrinsic themes expressed by the subjects were “hope of recovery”, “ambivalence about the future”, and “accept pain to become pain-free”. The first two themes were present when the subjects expressed their relationship to pain and when they described their thoughts about possible treatments. Often, a subject started with hope of recovery, followed by ambivalent feelings about the future, and then returned to hope. Subjects expressed similar themes when they described how they perceived the treatments and what the outcome of these treatments could be.

The subjects’ ambivalence about the future was also related to the treatment process, which involved painful procedures followed by periods without pain, and then the return of pain. This resulted in ambivalence about the future and whether there was hope of recovery beyond the painful treatments. In some cases, ambivalence about the future was related to the subjects’ prior treatments that had not improved the pain.

The theme “accept pain to become pain-free” was related to the service that was provided and was intrinsic in the subjects, and it was apparent in all of the interviews. The subjects described different levels of painful feelings during their procedures that ranged from “*...there was some physical pain...*” to “*...you could say it is like torture...*”. However, regardless of how painful the subjects described the procedures to be, they all described the pain as being worthwhile. They also elaborated by describing how they felt safe and calm during the encounters and that they had gained knowledge from the information and the explanations given by the healthcare team.

“*When you get better, it is worth all the pain, although you get frustrated when you need to test and test and test before you find out where the pain is coming from.*” Female, 52 years.

“*And then of course, it is painful when they are close to the nerves with a needle … ...But that pain is worth it every day of the week!*”

“*Although it is very painful, I can handle it because the environment is calm.*”

“*It was painful and unpleasant… I can’t see and I don’t have control… very exasperating. ‘Should I do this several times? Oh my God!’ I was thinking… it was unpleasant and I was afraid but I felt safe… petting the therapy dog during the injections made me calm… I am very afraid of syringes… I want to continue all the way even though it is unpleasant.*”

### Healthcare-focused themes: the importance of caregivers

Themes categorized in the group regarding the importance of caregivers were “availability for questions”, “previous caregivers’ ignorance and prejudice”, and “feeling safe”.

The first two themes were only expressed by women. They expressed frustration regarding the difficulty of getting in contact with the clinic they had previously been in contact with, resulting in the theme “availability for questions”. When they had questions, it was not possible to reach the clinic or the physician. This was expressed in contrast to their experiences at our clinic.

“*I was shocked that a doctor answered, that you were able to talk to a doctor as first contact… yes, I have had other tests at the hospital. They did something, but I don’t know what, and they implanted a dorsal column stimulator… when you go to an ordinary doctor you never know who it will be.*”

“*You can always call them… it has worked well… when I have had pain between encounters, I could call them and get explanations… they are available all the time.*”

However, their main frustration, especially among the women, was about “previous caregivers’ ignorance and prejudice”. The subjects said that physicians had negated their pain because their X-ray images and magnetic resonance imaging looked normal, and thus their pain was attributed to psychological distress. The subjects also mentioned that, when they asked to be referred somewhere to treat their pain, they were denied because the physician thought that treatment would be meaningless and ineffective.

“*I have been discredited so many times” … “They say my pain is because of my obesity.*”

“*I have struggled for so many years, but I have not gotten any help from the healthcare system… he had decided that nothing works and that’s it!*”

The theme “feeling safe” described our subjects’ experience of a stress-free environment and feeling they were listened to. They also mentioned that they could relax and remain calm, and that their confidence arose from their caregivers’ holistic approach. This was emphasized when they described the process of tests, assessments, and new tests, which started with the diagnostic procedures, and how this continued with them getting better, but the pain returned and new diagnostic procedures were performed until the diagnosis was set and treatment was given. They described the importance of the scheduled follow-ups and that they knew that they could return to the clinic if the pain returned.

“*It is difficult to describe, but it felt… well… like a good encounter*” … “*You felt calm and safe and received very good information.*”

“*They ask about the whole picture.*”

“*As I said, I am very scared of needles*” … “*Despite it being painful*” … “*As calm and pleasant as it could possibly be in this kind of situation.*”

“*You knew it was only a test, so you knew it would return, but then you really felt how bad the pain was… it is frustrating that you have to test several times before finding the right nerve… it is extremely painful, and you get shocked by how painful it is… it is like torture you could say; it is for sure, but it is so worth it, that it is.*”

### Outcome-focused themes

The theme “gain knowledge and understanding” was partly related to the services received and partly related to the outcomes, and described how the subjects felt that they were informed and how different aspects of their problems were explained so that they understood their pain. This was also connected to their general feelings of safety and their improved health.

“*I felt that I was seen… more than I previously felt from a doctor...then I gained more understanding of where the pain came from; it was so apparent for me… and I started to understand how much pain I had.*”

“*Now I can separate it more so I can understand it.*”

“*First of all, the doctor informed me how the body works*” ... “*This is very, very important! If you understand the context then you can understand your problem better… now I have an understanding of how one problem can be caused by problems in another part of the body. I understand my back better.*”

All of the subjects said that the reason for them seeking help after receiving previous care was that they still longed for a reduction in their pain level and for improved quality of life. However, these goals were not in focus when they described their experiences, they just mentioned them. They focused more on the outcomes and what they felt was the result of going through the program.

Although the subjects did not mention the goals often, and never elaborated upon the goals, they were eager to describe how their life had been changed as a result of the treatments. This theme, “improve health – a new life”, was not described as being a result of the reduction of the pain, but rather a result of the manner in which they were approached by the physician. The theme was also described as being related to the feeling of safety, the availability for further questions, and the knowledge they had gained.

“*I don’t want to die anymore, that is quite obvious… I have been seen and strengthened as a person… when I say that I don’t want to live anymore, he (the doctor) says that it is understandable; I have been normalized, I have been understood.*”

“*I was able to throw away all the pills I had been taking when I came to the doctor; my quality of life has improved immensely, because you want to have a life even if you still have pain.*”

The theme “help others” was clearly expressed as a result of their own improved health, and the subjects said that other subjects with pain should be provided with an opportunity to receive similar treatment. Several subjects mentioned that they had reached out to other people with pain who they knew and tried to persuade them to seek help and to be referred to the clinic.

“*It is a pity that not more people get the opportunity; I do everything I can to tell others about this.*”

“*I have told them about this at my work. So many of my coworkers have problems with back pain, so I mention this several times each year to people who might be searching for help.*”

### Disparities in themes according to age, previous experience, and gender

We did not find any differences in the occurrence or expression of feelings in the different themes when we divided the subjects by age, how long they had suffered with pain, and whether their health was improved by their treatment. When the subjects described procedures and treatments and how they felt, they related to the treatments. Those with experience of IPM performed at other units did not relate specifically to the treatments received at this ward. The only gender difference we could see was only women expressed the themes “availability for questions” and “previous caregivers’ ignorance and prejudice”.

### Empowerment

In summary, the transcribed interviews show that the subjects’ perceptions shifted among three key themes. First, there was themselves as objects, including the treatment they received, how they felt, and their fears. Second, were the caregivers, including tests and retests, the encounters and interactions with the professionals, and the availability of help. Third, were the outcomes, including the results of the tests and treatments, and how the outcomes inspired the subjects to say that other people with pain should be offered the same opportunity. The subjects partly said this to emphasize how much they value the care, partly to demonstrate frustration with waiting for so long themselves to get help, and partly to express a genuine feeling for other subjects with pain. Linking these themes, the subjects expressed something best described as “gained empowerment” during IPM. They described they felt being heard and seen, gaining knowledge that helped them understand their problem better, being able to ask questions and receive answers, and feeling safe and listened to.

### Confirmation of results

After the analysis, eight of the subjects were contacted by the last author (K-GS); the other subjects could not be reached. These eight subjects confirmed the results and stressed that “*…it is very emotional, that we all seem to have the same experiences*” or “*I have nothing to add; it is amazing how well you have understood me*”.

## Discussion

### Framework for the analysis

**Fig. 1:**
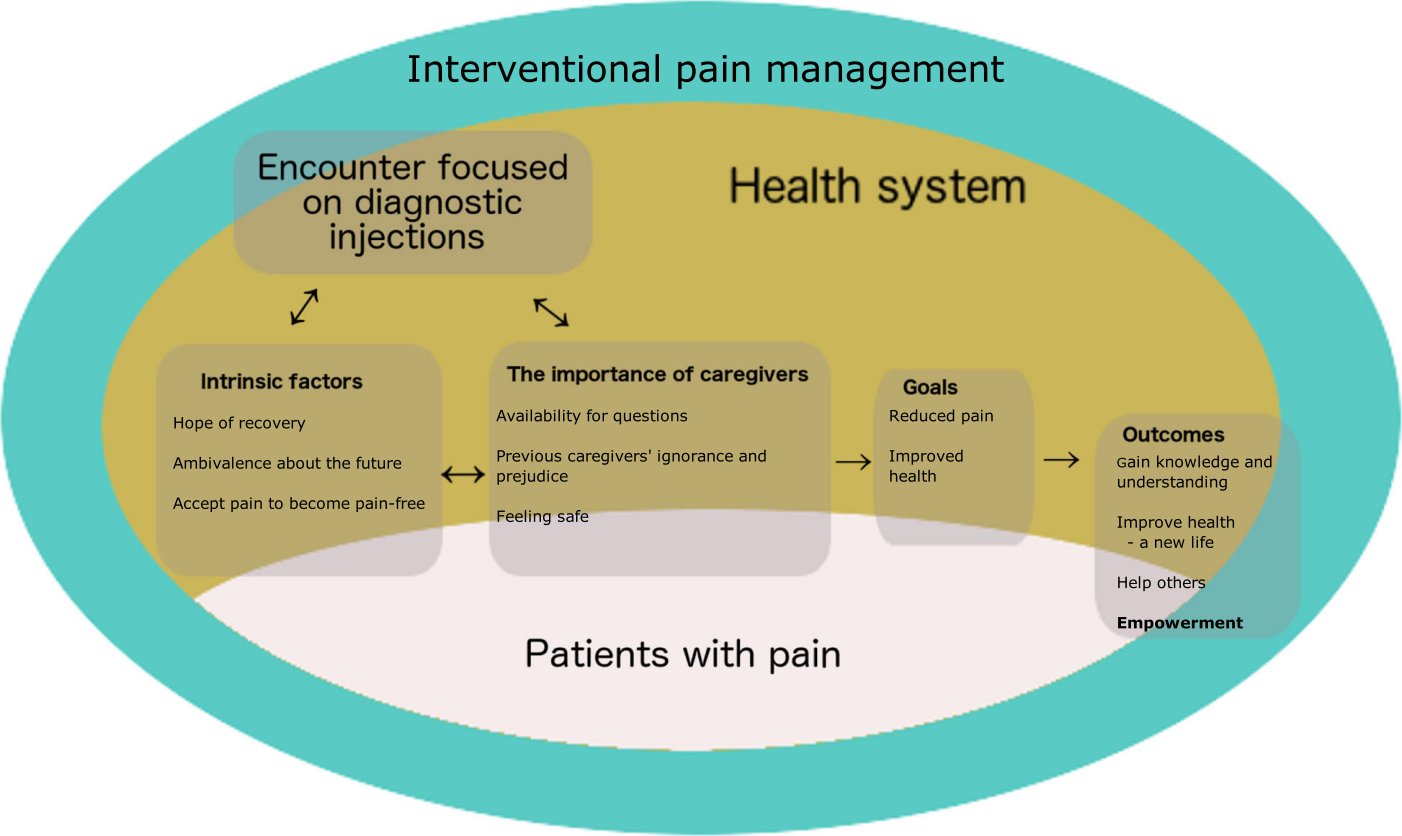
Themes identified in this report and how they relate to each other

We analyzed interviews with subjects who attended our clinic for IPM with the aim of understanding more clearly what they feel and the reasons for the apparent changes we have seen. We found some themes we expected to find, while other themes were new to us.

van Olmen et al. [38] presented a model of the interactions between different parts of the healthcare system that visualizes the context and role of leadership together with its relation to the outcomes and goals of the healthcare. This model has been used to analyze weaknesses in healthcare systems and to find ways to strengthen such systems [44]. We found that, by adopting parts of the model we could use it to describe how the themes were related to each other and to clarify the roles of the themes (Fig. 1). By exploring the IPM encounters through the themes identified here and examining the themes as a micromodel of a healthcare system, we identified the different elements and how they are related. Similar to how the quality of a healthcare system depends on how well it corresponds to the needs of the population, the quality of IPM depends on how well the subjects’ needs are met [45, 46].

### Identified themes

Laying out the themes showed that the subjects possessed the same basic values as the general population, and the themes evolved from their contact with the healthcare services. The services received by the subjects included some inherent themes, such as the need to accept painful tests to become pain-free, but the themes were mainly dependent on the subjects’ contact with their healthcare providers (i.e., the encounter with the physician and the information/explanation given by the physician). The outcomes are the result of these interactions more than the result of reaching the primary goals, (i.e., become pain free). This interaction became clearer when the themes were applied to the original interviews. Themes derived through interactions between two different elements are often based on codes found in close proximity to another in the interview; for example, the theme “improved health” was closely connected to the themes “gain knowledge and understanding” and “feeling safe”.

### Altruism as a sign of improvement

There is an old debate about the nature of humans as being universally selfish or universally good, and there is evidence that a person’s position on this scale changes with time as a sign of neuronal plasticity [47]. This repositioning is apparent in how the subjects described their experience of IPM in the present study. Initially, they were focused on their own pain and suffering, and their previous experiences and their need for help. However, as they began to relax, feel safe, and gain knowledge and understanding, they let go of their struggle for themselves and started to think about other people with pain who they knew and hoped that they might get help similar to themselves. Such a sentiment is often expressed by subjects met by caregivers in an IPM clinic. At the end of an encounter, just before they leave, the question “*By the way, could this help with XXX? My ZZZ has had problems with...*” is often posed. This type of question is common and emphasizes that pain is central to our consciousness [48].

### Empowerment

When the themes were merged and structured into the model, a cohesive pattern of empowerment appeared. The concept of empowerment, where individuals gain mastery of their own affairs, has become more important in recent years [49]. In this study, we saw how the subjects described prosperity, hope, and increased knowledge and understanding, and how they felt safe. The subjects reported that the pain experienced during the treatment was worthwhile because they understood that it is part of the process of tests, assessments, and new tests and might result in improved health. Their thoughts about helping others gain access to similar care represented their increased capacity to make choices and to transform these choices into actions [50].

Trying to understand the interconnections among the themes was a process similar to trying to understand how a disease control program connects to the general healthcare system [44]; in other words, we took the process’s perspective to understand how the process connects to the patient’s experiences [51]. This approach allowed us to deduce aspects beyond the individual that apply more generally to IPM in this setting [52]. Empowerment depends on the individual’s ability to remember and process their experiences, and thus a treatment that affects these abilities, for example, a medication that causes ante-grade amnesia, might reduce patient empowerment [53–55].

### The interviews

This study was performed using telephone interviews to gather information from subjects living far from the clinic. There are several challenges with telephone interviews compared with face-to-face interviews [56]. There is a risk of losing the subject’s involvement, although this was addressed by selecting the subjects during a face-to-face meeting with the nurse. When listening to the recordings before they were transcribed into text, we felt that this was not a problem in this study. However, the risk that subjects were indecisive about providing answers to questions remains. Being interviewed by telephone by an interviewer with whom they had no prior relationship may have made the subjects more comfortable and minimized the risk that the reactions of the interviewer would influence the responses. Communication with subjects that provides extraneous information is challenging because this information may be lost over the telephone. However, in the pilot study, the extraneous information tired the subjects, so they did not have the energy to finish the interview. Thus, the limits inherent in telephone interviews also facilitated focused information exchange. There was also a clear risk of communication with a third party, but because we recorded the interviews, we could check that there were no third parties who spoke. It is impossible to know whether there were other people available in the room during the interview, but were no signs that this was the case. In summary, the decision to perform the study via telephone interviews instead of face-to-face interviews may have filtered out some information we would have gained during a face-to-face interview, but we think that the benefits of telephone interviews were greater and they provided information that would not otherwise be accessible.

### Diversity

A qualitative study aims to identify as many aspects of the subjects experiences as possible in a given situation, population, or treatment. We managed to enroll a wide range of patients in terms of age, gender, and pain duration. However, the study was performed in a single clinic for interventional pain management, so we might have overlooked aspects of the topic because we did not include a wide range of health-care providers. However, those who also had experience of IPM at other clinics presented their experiences as more a result of the technique than of the encounter at the studied clinic.

### Empowerment in pain management

The importance of giving patients more choice and control over their care is often emphasized [57]. Nevertheless, many patients describe feeling disempowered when seeking help for their chronic pain [22, 29]. This was also apparent among the subjects in this study who had previously been through a PRP. Interventional pain management is based on the fact that pain generators cannot be localized by diagnostic imaging [58–60], thereby forcing the provider to cooperate closely with the patient. For example, a diagnostic nerve-block is performed under X-ray control and the patient evaluates the effect on the pain level compared with before the injection [38]. A diagnosis could not be established without this cooperation, and as a side-effect, the patient is involved in the process and gains control over their care. However, we found no study that explores the patients’ experiences of interventional pain management.

### Generalization

The empowerment described by the subjects in this study could be an effect arising from the favorable outcome of the treatments. However, when the interviews were performed, only nine subjects reported a positive effect on their HRQoL. Therefore, a more relevant question is whether the empowerment is a result of the interventional approach and can be generalized or if it is a result of the positive attitude and personal qualities of the caregivers in the clinic. Although some of the subjects described the caregivers in positive terms, the descriptions were mainly related to the treatments and the interventional approach. This was especially apparent among those who also had experience of IPM at other clinics. Therefore, we believe it is possible to generalize the findings cautiously.

## Conclusion

In this study, we have started to reveal some of the psychological aspects of IPM, and we hope that our findings might provide a step towards a more integrated approach between the interventional and rehabilitation-focused communities because many patients with chronic pain need both kinds of treatment. The subjects shared the basic values of the general population, although many of the themes evolved in relation to the contact with the healthcare services they received. When the themes were merged and structured into the model, a cohesive pattern of empowerment appeared. Nevertheless, further patient-focused qualitative studies at other pain management clinics (both rehabilitation clinics and interventional clinics) are needed to further explore the role of empowerment. If empowerment is a major contributor to the positive effects of IPM, it is important to facilitate and not hinder empowerment.

## Supplementary information

**Additional file 1.** Interview guide.

## Data Availability

The datasets generated and analyzed during the current study are not publicly available due to Swedish law but are available from the authors on reasonable request if given specific approval from the Regional Ethics Board in Umeå, Sweden.

## Abbreviations

IPM: Interventional pain management
PRP: Pain rehabilitation program
HRQoL: Health-related quality of life
EQ-5D-3: EuroQOL 5-dimensional 3-level survey
MBB: Medial branch blocks to diagnose zygapophyseal joint pain
LBB: Lateral branch blocks to diagnose sacroiliac joint pain
Phys: Physiotherapy
Med: Medication
NB: Preipheral nerve blocks
RF: Conventional radiofrequency denervation
pulsed-RF: Nerve stimulation with pulsed radiofrequency current
IL: Interlaminar nerve root block
TR: Transforaminal nerve root block
Fusion: Surgery with vertebral fusion
DCS: Dorsal column stimulation
ia: Intraarticular injections
CII: Continuous intrathecal opioid infusion
Symp: Nerve block of sympathetic nervous system
TP: Trigger point injections
