# Supplementary material for: Patient perspectives on interventional pain management: thematic analysis of a qualitative interview study
Source: BMC Health Serv Res. 2020 Jul 1;20:604. doi: 10.1186/s12913-020-05452-7 (PMC7329503; doi:10.1186/s12913-020-05452-7)
Supplement: Supplementary file 1 — Additional file 1. Interview guide. [file 12913_2020_5452_MOESM1_ESM.docx]

# Additional file 1. Interview guide

We would like to know about your experiences with interventional pain management. Therefore I would like you to tell me what you have felt and experienced during the management and what thoughts you have had. I will help you with extra questions during the interview.

What did you think when you were in the waiting room before the first visit?

Do you remember what you were thinking during the first encounter with the doctor, when he posed the questions?

How did you experience the first test injections? Have you had similar experiences before?

What did you feel immediately after the injections when you got up from the table?

Was your pain affected by the injections? Was it reduced?

Do you remember what you thought when you left for home after the first encounter?

When the pain returned, what did you think? How did you feel?

An investigation means that you start with pain, get an injection and the pain is reduced, then the pain returns again and this is repeated several times. What was your experience of this?

Has your understanding of your problems changed in any way? If so, how?

Has the investigation led to a radiofrequency denervation? What was your experience of this? Do you remember what you thought before the first time that was done? How did you feel afterwards? Were you very affected by the treatment? Did you get better because of the treatment? If so, how long did this improvement last?

Do you think you have changed as a person by the investigation? Has your relation to your pain changed? If so, how?

Many of the test injections and treatments are painful. And sometimes the pain is reduced after the injections, but sometimes not. And sometimes the improvements only last for a short while. If you had a friend who was going to be examined, how would you describe your experiences?
